# Antibiotics and over the counter medication use and its correlates among Arab pregnant women visiting a tertiary care hospital in Riyadh, Saudi Arabia

**DOI:** 10.12669/pjms.332.12376

**Published:** 2017

**Authors:** Hafsa Raheel, Sulaiman Alsakran, Abdulkhaliq Alghamdi, Majed Ajarem, Salman Alsulami, Afzal Mahmood

**Affiliations:** 1 Dr. Hafsa Raheel, Associate Professor, Department of Family & Community Medicine, College of Medicine, King Saud University & King Khalid University Hospital (KKUH), Riyadh, Saudi Arabia; 2 Sulaiman Alsakran, King Saud University & King Khalid University Hospital (KKUH), Riyadh, Saudi Arabia; 3 Abdulkhaliq Alghamdi, King Saud University & King Khalid University Hospital (KKUH), Riyadh, Saudi Arabia; 4 Majed Ajarem, King Saud University & King Khalid University Hospital (KKUH), Riyadh, Saudi Arabia; 5 Salman Alsulami, King Saud University & King Khalid University Hospital (KKUH), Riyadh, Saudi Arabia; 6 Dr. Muhammad Afzal Mahmood, School of Public Health, University of Adelaide, South Australia

**Keywords:** KEY WORD: Pregnant women, Saudi Arabia, Riyadh, Over the counter medications

## Abstract

**Background and Objective:**

The use of over the counter medications (OTCMs) is a common practice globally. OTCMs are of special concern among pregnant women as they pose risk to the mother and fetus. The objective was to assess the use of OTCMs by pregnant woman, and factors associated with it.

**Methods:**

A cross-sectional study, using a structured self-administrated questionnaire was conducted at the obstetric and antenatal clinics at King Khalid University hospital, Riyadh. 354 pregnant women were surveyed.

**Result:**

About 32% of the study participants used OTCMs without prescription at least once during their last pregnancy. The most commonly used OTCM was Paracetamol (22%). Pharmacists were the main source of knowledge (53%) for the participants. Correct knowledge regarding the drugs was associated with the usage of OTCM drug.

**Conclusion:**

Use of OTCMs and antibiotics during pregnancy is a common practice among Saudi pregnant women. General awareness regarding OTCMs use during pregnancy without prescription needs to be raised in the community. Pharmacists and media can help in providing accurate knowledge.

## INTRODUCTION

The world health organization (WHO) has described over the counter medication (OTCM) as the selection and use of medicine by individuals to treat self-recognized illnesses or symptoms.^1^ Non-prescribed usage of OTCMs is a common worldwide phenomenon.^2^,^3^ About 89% of the pregnant women in US, were reported to be using OTCMs at least once during their pregnancies.^3^ A high prevalence has also been reported in the European countries; 95% in the Netherlands, 93% in Iceland, and 92% in Finland as well as in Nigeria and Pakistan.^4^-^6^

Most common drugs obtained by pregnant women over the counter are paracetamol, ant-acids, anti-allergy and antibiotics. It is debated that usage of OTCMs lessens the burden of patient visit to the GPs, and on the health care system. However, when OTCMs, without prescription are used by pregnant women, it is of concern. Drugs used during pregnancy should be with caution. Many researches have raised concerns over the use of OTCMs during pregnancy.^7^,^8^

To our knowledge there is little information about OTCMs use by pregnant women in KSA. A study conducted in Taif,^9^ in 2013 has shown that medication usage during pregnancy was 40%. However, it looked at medication usage upon prescription, also its findings are not generalizable to all the Saudi population. We hypothesized that OTCM usage without prescription would be high among pregnant women in Riyadh. We also wanted to access the factors that are related with drug usage without prescription in the pregnant females of Riyadh, so as to formulate public health programs targeting practitioners, pharmacies, and women for awareness and change in practice.

## METHODS

We conducted a cross-sectional, self-administered, structured questionnaire study at the obstetric and antenatal clinics at the King Khalid university hospital (KKUH), King Saud University Riyadh.

King Khalid university hospital (KKUH) is a teaching University hospital, providing primary, secondary and tertiary care, in the city of Riyadh. It also provides care to patients referred from other hospitals as well as from other cities of the Kingdom. All care and medication is free of charge.

Women visiting the antenatal and obstetric clinics of KKUH were approached. All females attending and waiting, in the waiting room were offered to participate in the survey. We included pregnant Saudi women who were currently living in Riyadh and excluded women who were not pregnant at that time, living outside Riyadh.

### Sample size

A sample size of 322 was calculated. We added 10% to this, keeping in mind the non-response rate. So the final sample that we collected was 354.

n = (Za/2)2 p(1-p) / d2

(1.96)2 (.7(.3)) / (.05)2 = 322 pregnant women

### Data collection method

A structured closed ended questionnaire was formulated. It had four sections: socio-demographic, knowledge, attitude, and practice section. In the knowledge section they were asked about awareness of risk of specific drugs. Attitude section consisted of statement for pregnant women to answer. Practice section contained the drugs that were used by pregnant women. Pregnant women were asked to report OTCMs usage.

Piloting was done on 10% of the total sample, to pick up ambiguities. The questionnaire was revised based on the results of the piloting. Data entry and analysis was done using statistical package of social sciences (SPSS) version 21. Simple descriptive analysis and cross tabs were done. Crude odds ratio and multivariate model was run.

### Ethical issues

IRB approval was obtained from the Medical College Board at the King Saud University. Clear and detailed information was provided for informed consent, and the women were reassured about their right to participate or withdraw from this study without any fears about access to care or quality of care. No personal identifying information was collected (name, address), and information about individual women was reviewed only by the researcher.

## RESULTS

Total 354 pregnant women were enrolled in the study. Three hundred twenty two(91%) pregnant women completed the survey and 32 women didn’t complete the survey. The sociodemographic characteristics are shown in Table-I.

**Table-I:** Table Showing the Personal and Demographic Characteristics of Participants of the Study on OCTMs usage during pregnancy in Riyadh

*Variables*	*n=322 (%)*
***1. Age***	
<20	8 (2.5)
20-30	217 (67)
31-41	82 (25.5)
>40	15 (5)
***2. Nationality of participants***	
Saudi	294 (91)
Non-Saudi	28 (9)
***3. Education of the participants***	
Elementary	9 (3)
Intermediate	15 (5)
High school	114 (35)
Bachelor	162 (50)
Higher education	22 (7)
***4. Occupation of the participants***	
House-wife	171 (53)
Student	45 (14)
Health-related employee	14 (4)
Teacher	52 (16)
Others	40 (13)
***5. Approximate monthly income***	
<3,000	15 (5)
3,000-4,999	66 (20)
5,000-9,999	152 (47)
10,000-20,000	77 (24)
>20,000	12 (4)
***6. Number of Pregnancies (current and previous)***	
1	137 (43)
2	59 (18)
3	49 (15)
4	26 (8)
>4	51 (16)
7. Disease*	
No disease	200 (62)
Diabetes	28 (9)
Hypertension	8 (2.5)
Asthma	25 (8)
Hypothyroidism	25 (9)
Anemia	42 (13)
Others	32 (10)

* multi-response table

**Fig.1:**
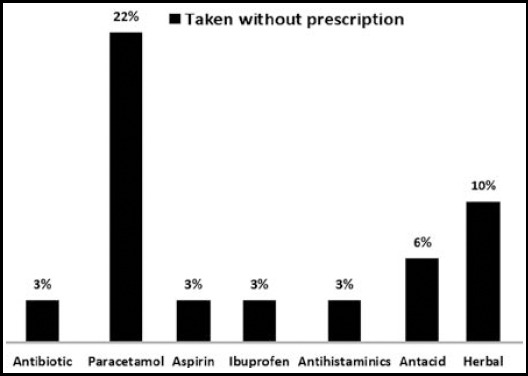
Figure showing medications that have been obtained from pharmacies by the study participants during pregnancy.

About 32% of the respondents had used a drug at least once during their pregnancy. Fig.1 was multiple response table shows the most frequently obtained drug over the counter. Antibiotics and paracetamol were the commonly obtained OTCM with prescription. While paracetamol and herbal medicines were the most frequently obtained without prescription.

Overall about 50% of the participants were aware that drugs during pregnancy should be used with prescription. However, about 90% of the respondents were of the opinion that if they are sick during pregnancy, they would go directly to the pharmacists and obtain drugs without doctor’s prescription. This was despite 85% were of the opinion that OCT drugs are harmful to the fetus.

The source of information regarding OTC drugs were pharmacists (53%), medication pamphlets (28%), media (27%), family member (11%), and friends (4%). The response was taken as multiple response. Bivariate analysis was run to find out the factors associated with usage of OTCM during pregnancy. The results are displayed in Table-II. No significance association was found between sociodemographic variables and the OTCM usage. Participants who were knowledgeable regarding herbal medications usage and aspirin usage without prescription were more likely not to use OTCM without prescription. (OR 1.65, CI 1.0 -2.7, OR 1.78, CI 1.0-3.1). However, participants who were not knowledgeable regarding antacid safety and usage during pregnancy, where more likely to use OTCs without prescription (OR 1.9, CI 1.1 – 3.0).

**Table-II:** Table showing the factors associated with OTC drug usage.

*Factors*		*OTCMs use during pregnancy*		*RR*	*95% CI*

		*Yes n=103 (%)*	*No n=219(%)*		
Age	Age =< 30	71(69)	154(70)	1.06	0.64-1.77
	Age > 30	32 (31)	65 (30)
Education	Women with lower education	49 (48)	89 (41)	1.32	0.82-2.12
	Women with higher education	54 (52)	130 (59)
Occupation	House wife	54 (52)	117 (53)	1.04	0.65-1.66
	Employee women	49 (38)	102 (47)
Income	Low income family	31 (30)	50 (23)	1.45	0.86- 2.46
	High incomefamily	72 (70)	169 (77)
Pregnancy	First pregnancy	41 (40)	96 (44)	1.18	0.73- 1.90
	Multiple pregnancy	62(60)	123 (52.5)
Knowledge About drugs	Not knowledgeable	46(45)	86(39)	1.24	0.77- 2.0
	Knowledgeable	57(55)	133(61)

## DISCUSSION

To our knowledge this is the first study in Saudi Arabia that focused on the use of OTCMs by pregnant women. The study pointed to some important findings relevant to the health of pregnant women and their babies. Compared to the high percentage of women using OTCMs in the US and European countries,^3^,^4^ about 32% of women in our study stated that they used OTCMs at least once during their pregnancy. This could be an under reporting, as we noted that many of our study participants had the knowledge that OTCMs during pregnancy may be harmful. Also we fear that some participant’s in our survey may not have reported using OTCMs considering that the survey was in hospital setting and participant’s may have felt that if they acknowledged using the OTCMs they may be judged negatively by the care providers. On the other hand, considering the fact that access to the health system, and physicians is not a difficult process in Riyadh affordability of health care in not an issue, heath care being totally governmental issue, is free for all state citizens. There is no fee for consultation in the public sector, we were surprised that females were visiting drug stores and obtaining medication without consultation with physician.

Surprisingly, none of the sociodemographic characteristics, age, education, economic status, neither knowledge regarding the drugs were found to be significantly influencing the usage of OTCMs. This is in contrast to the finding of a study in Nigeria^10^ which showed that OTCM usage during pregnancy was more among self-employed or unemployed females, and the multigravida females. Similarly Lupattelli et al.^2^, in their multinational web based study reported that older aged women, housewives, less educated women, and women with unplanned pregnancy were more likely to use OTCMs. We did find that those women with less, or incorrect knowledge on drug usage during pregnancy, were more likely to be using the OTCMs. This was also reported by other studies.^5^,^6^

Studies done globally have reported paracetamol, herbal medication, and vitamins to be the major over the counter drugs obtained by pregnant women during pregnancy.^4^-^6^ Our study also pointed to such use. In addition to paracetamol, antacid and herbal medicines, the study also pointed to the use of antibiotics without prescription. Dispensing of antibiotics without prescription is a known concern in Riyadh, as Bin Abdulhak 2011 study, surveying 327 pharmacies in Riyadh city alerted to the fact that antibiotics without prescription were dispensed in 78% of the sample of pharmacies that were surveyed. Majority were dispensed on simulated cases of sore throat and urinary tract infection.^11^ The use of antibiotics reflects a lack of emphasis in ANC services on providing education to the pregnant women about the potential negative effects of antibiotics on mother and baby. Such use of antibiotics by pregnant women without prescription is alarming, requires further investigation at a larger scale and formulating appropriate health promotion messages and measures.

Another matter of concern is that many respondents were reported using herbal medication. Little is known about the potential benefits or hazards of herbal medication usage during pregnancy. Further research on what type of herbal medication was used, extent, and effects, side effects during pregnancy needs to be conducted. It would be useful to compare the pregnancy outcomes among those who used herbal medicines and those who didn’t.

For the majority, the main source of information regarding drug use was pharmacists. This again differs from studies conducted elsewhere where husbands and family members were reported to be main source of information.^5^,^6^ It could be a reason that in KSA pharmacists are often well regarded by the community with many considering the pharmacists as physicians. It is mandatory by Kingdom law that all pharmacies, private or governmental should be run by a pharmacists at the dispensing counter. This makes it understandable why they were the main source of information for these pregnant women. Similar findings were reported by Abeje et al.^12^ who reported that women whose homes were far away from the health facilities, were more likely to be approaching pharmacies directly and obtaining medication without prescription. The study participants also relied on the medical pamphlets and media for knowledge regarding the information.

### Limitations of the study

Out study had the inbuilt problems of a quantitative study. Some aspects of attitudes of the respondent and knowledge were explored in detail. Also the reason as to why we could not find association between socio-demographic variables and OTCM use could be related to the inherent problems of a cross sectional study design. This study was conducted in Riyadh, capital of the Kingdom, so results cannot be generalizable to the rest of the country. Saudi Arabia being a large country have a mix of urban and rural settings and therefore, these finding could be quite different from other areas. Also we did not look into the reasons why pregnant females were obtaining OTCMs. We recommend that further studies conducted explore the root causes of using OCTMs.

### CONCLUSION

Our study therefore highlighted that OTCMs is a common phenomenon in the Saudi community. Pregnant females are taking medicines without prescription and their main source of knowledge are pharmacists, medical pamphlets, and media. Antibiotics are also being obtained without doctor’s prescription. This points to the fact that they are unaware of the potential hazards of the antibiotics on pregnancy and the fetus.

### Recommendations

We can recommend that media can be utilized as a potential information provider to the masses. Drug safety information needs to be raised in general. Policy needs to be placed so that Pharmacists take clear and detail history before dispensing any medication.

## References

[1] Black RA, Hill DA (2003). Over-the-counter medications in pregnancy. Am Fam Physician.

[2] Fingleton NA, Watson MC, Duncan EM, Matheson C (2016). Non-prescription medicine misuse, abuse and dependence:a cross-sectional survey of the UK general population. J Public Health (Oxf).

[3] Mitchell AA, Gilboa SM, Werler MM, Kelley KE, Louik C, Hernandez-Diaz S (2011). Medication use during pregnancy, with particular focus on prescription drugs 1976- 2008. Am J Obstet Gynecol.

[4] Lupattelli A, Spigset O, Twigg MJ, Zagorodnikova K, Mardby AC, Moretti ME (2014). Medication use in pregnancy:a cross-sectional, multinational web-based study. BMJ open.

[5] Bohio R, Brohi ZP, Bohio F (2016). Utilization of over the counter medication among pregnant women;a cross-sectional study conducted at Isra University Hospital, Hyderabad. J Pak Med Assoc.

[6] Yusuff KB, Omarusehe LD (2011). Determinants of self medication practices among pregnant women in Ibadan, Nigeria. Int J Clin Pharm.

[7] Eyers S, Weatherall M, Jefferies S, Beasley R (2011). Paracetamol in pregnancy and the risk of wheezing in offspring:a systematic review and meta-analysis. Clin Exp Allergy.

[8] Nakhai-Pour HR, Broy P, Sheehy O, Bérard A (2011). Use of nonaspirin nonsteroidal anti-inflammatory drugs during pregnancy and the risk of spontaneous abortion. CMAJ.

[9] Zaki NM, Albarraq AA (2014). Use, attitudes and knowledge of medications among pregnant women:A Saudi study. Saudi Pharm J.

[10] Kebede B, Gedif T, Getachew A (2009). Assessment of drug use among pregnant women in Addis Ababa, Ethiopia. Pharmacoepidemiology Drug Saf.

[11] Bin Abdulhak AA, Al Tannir MA, Almansor MA, Almohaya MS, Onazi AS, Marei MA (2011). Non prescribed sale of antibiotics in Riyadh, Saudi Arabia:A Cross Sectional Study. BMC Public Health.

[12] Abeje G, Admasie C, Wasie B (2015). Factors associated with self medication practice among pregnant mothers attending antenatal care at governmental health centers in Bahir Dar city administration, Northwest Ethiopia, a cross sectional study. Pan Afr Med J.

